# Analysis between symptoms of the upper gastrointestinal tract and endoscopic findings of patients undergoing upper digestive endoscopy in a reference center in the interior of Maranhão, Brazil

**DOI:** 10.1590/acb395824

**Published:** 2024-09-30

**Authors:** Luis Thadeu Rebouças Santos, Mariana Paiva Braga Martins, Caio dos Santos Souza, Rodrigo Rodrigues Silva, Marcos Antonio Custódio Neto da Silva

**Affiliations:** 1 Universidade Federal do Maranhão – Imperatriz (MA) – Brazil.; 2 Hospital Macrorregional Ruth Noleto, Serviço de Endoscopia – Imperatriz (MA) – Brazil.; 3 Universidade Federal do Maranhão – Centro de Ciências – Imperatriz (MA) – Brazil.

**Keywords:** Dyspepsia, Endoscopy, Helicobacter pylori

## Abstract

**Purpose:**

To analyze clinical and endoscopic aspects of dyspeptic patients submitted to upper endoscopy in a reference center in the interior of Maranhão, Brazil.

**Methods:**

Observational, descriptive, and analytical research through interviews and endoscopic reports of 80 patients with dyspeptic complaints submitted to upper endoscopy.

**Results:**

Among the respondents, 66.25% were women, most were aged ≥ 40 years old and had epigastric pain as their main symptom, and 29.75% had no appropriate indication to perform upper endoscopy. Mild enanthematous gastritis of the antrum was the most frequent finding, and 92.5% had non-significant findings. Rapid urease test was positive in 25%. The following findings showed a statistically significant correlation (*p* < 0.05): age < 40 years old, female gender, and gastric lesion with positive urease test; smoking with gastric lesion and age less than 40 years old with normal examination. Patients with significant findings had appropriate indications for upper endoscopy.

**Conclusions:**

The correct indication of upper endoscopy is essential for satisfactory endoscopic yields and accurate diagnosis.

## Introduction

Dyspepsia or dyspeptic syndrome can be characterized by a set of symptoms related to the upper digestive tract, such as epigastric pain, postprandial discomfort, early satiety, retrosternal pyrosis, regurgitation, and heartburn^1^. In this context, according to the Rome IV criteria, this syndrome can be classified as functional or organic. Organic dyspepsia is related to confirmed structural or histopathological changes, such as peptic ulcer disease, gastroesophageal reflux disease (GERD), gastric cancer, gastritis, and *Helicobacter pylori* infection. Functional dyspepsia, on the other hand, is defined by symptoms unrelated to structural disease, and upper gastrointestinal endoscopy (UGE) findings demonstrate normality or changes not compatible with the symptoms^2^.

Diagnosis and therapeutic management in these cases are essentially clinical, requiring complementary tests depending on the indications. UGE can be used to provide information for diagnosis and staging, differential diagnosis, investigation of complications, and treatment of gastrointestinal disorders^2^^,^ 3. In this context, according to the IV Brazilian Consensus on *H. pylori* Infection, UGE should be performed in patients aged 40 years old or older with uninvestigated dyspepsia, patients who do not respond to empirical treatment with histamine H2 receptor blockers (H2), proton pump inhibitors (PPIs), or prokinetics, among others, and patients of any age with alarm signs, including unintentional weight loss, dysphagia, persistent vomiting, palpable abdominal mass, jaundice, bleeding, and a positive family history of gastric cancer in first-degree relatives^4^.

The prevalence of dyspepsia is high worldwide, with an estimated rate of 10 to 30%^5^. In line with this, gastroesophageal reflux disease, one of the main causes of dyspepsia, motivates more than 5.6 million medical consultations each year^6^.

Considering the above, the present study aimed to analyze the clinical and endoscopic aspects of dyspeptic patients undergoing UGE at a reference center in the interior of Maranhão, Brazil.

## Methods

### Study type

This is an observational, descriptive, and analytical study of patients with dyspeptic complaints undergoing UGE at the Digestive Endoscopy Service of the Macrorregional Hospital Dra. Ruth Noleto in Imperatriz, Maranhão.

### Inclusion and exclusion criteria

All patients aged 18 years old or older, of both sexes, with symptoms of the upper gastrointestinal tract, referred for UGE, and who agreed to participate in the research by signing the informed consent form (ICF) were included. Exclusion criteria were any indication for UGE other than dyspepsia, the use of antacids or gastric secretion inhibitors in the two weeks prior to endoscopic examination, pregnancy or lactation, age under 18 years old, surgical conditions such as vagotomy, previous gastric resection surgery, or pyloric stenosis. Additionally, those who did not sign the ICF were excluded.

### Data collection

Clinical data were collected through an interview with the volunteer at the research site before undergoing endoscopic examination from October 2022 to February 2023. Data were filled in a semi-structured questionnaire with clinical and endoscopic variables of the patients included in the study. Variables collected included name, gender, age, clinical presentation, comorbidities, risk factors, history of previous endoscopies, and whether they had appropriate indications for UGE according to the IV Brazilian Consensus on Heliobacter pylori Infection.

Regarding risk factors, a family history of gastric cancer was defined as those reporting such pathology in a first-degree relative. Non-selective or high-dose use of nonsteroidal anti-inflammatory drugs, smoking, alcohol consumption, and daily coffee intake were considered as risk factors. The criteria for appropriate UGE indications were based on the IV Brazilian Consensus on Heliobacter pylori Infection.

Subsequently, the data were entered into a Microsoft Office Excel spreadsheet (365 version), in which the anatomical location of the lesion seen in UGE (esophageal lesion, gastric lesion, duodenal lesion, or normal examination) and the severity of the lesion were classified. The latter was categorized into significant or non-significant findings based on the classification by Wallace et al.7, defining a significant finding as the presence of ulcers, tumors, or strictures.

### Sample size calculation

To be a finite sample, since the number of cases is small in relation to the total population, we use a calculation formula for a finite population that corresponds to Eq. 1:


$$n=\frac{n_{0}}{1+\frac{n_{0}-1}{N}}$$ (1)

*n*_o_ = sampling intensity; N = sample size in the population.

Where *n*_o_ is equal to Eq. 2:


$$n_{0}=\frac{Z(K{)}^{2}}{4d^{2}}$$ (2)

where: z(K)^2^ = value in the Student’s t-test table with (n-1) degrees of freedom for the confidence interval; d = error.

Taking into account the research carried out by Oliveira et al.8, which found 1,730 cases, we have to:

Initially it was determined *n*_o_ (Eq. 3):


$$n_{0}=\frac{1,96^{2}}{4(0,05{)}^{2}}=384,16$$ (3)

Replacing *n*_o_, we have Eq. 4:


$$n=\frac{384,16}{1+\frac{\left(384,16-1\right)}{1730}}=315$$ (4)

If we understand that this work was carried out over a period of time corresponding to five months, taking into account the maintenance of case numbers, we will have a sample corresponding to 80 patients.

### Statistical analysis

In data processing, the database was initially imported from the Microsoft Office Excel spreadsheet program (365 version) to the open-access statistical program R Studio. Categorical variables were expressed in frequencies (n) and percentages (%). The association between two categorical variables was performed using the Pearson χ^2^ test or Fisher’s exact test, chosen based on the frequency of individuals in the cell. The statistical significance was established at *p* < 0.05.

### Ethical aspects

Data collection was carried out after the signing of the ICF. This study followed the guidelines of Resolution no. 466/12 of the National Health Council, and data collection began after approval by the Research Ethics Committee of the Universidade Federal do Maranhão. All collected information was for exclusive use of this research, with no other purposes. Data privacy will be guaranteed, and the researcher is responsible for organizing the data to comply with ethical aspects. The study was approved by the Research Ethics Committee of the Universidade Federal do Maranhão under the number 5.693.609 and approval number Certificate of Presentation of Ethical Review 57473522.4.0000.5086 on the Brazil Platform, following the guidelines of Resolution No. 466 of December 12, 2012, of the National Health Council. The translation of this work was carried out with the help of artificial intelligence (AI). AI, represented by the GPT-3.5 language model developed by OpenAI, played a fundamental role in the transposition and accuracy of scientific content into the target language.

## Results

### Sociodemographic and clinical data

Data from 80 dyspeptic patients who underwent UGE at Macrorregional Hospital Dra. Ruth Noleto, from October 2022 to February 2023, were analyzed. As shown in Table 1, most patients were female (66.25%). The most prevalent age group was between 29 and 39 years old (36.25%), and most respondents were aged 40 years old or older when stratifying the minimum age for UGE (53.75%). Epigastric pain was the most frequent symptom, reported by 77.5% of patients. Most patients had two symptoms (23.75%), followed by three symptoms (22.5%). During the interviews, 29.75% of patients did not have an indication for UGE according to established criteria.

**Table 1:** Presentation of clinical features in patients with dyspepsia undergoing upper endoscopy.

Variables		n	%
**Sex**	Female	53	66.25
	Male	27	33.75
**Age by age group (years old)**	18 to 28	8	10.00
	29 to 39	29	36.25
	40 to 50	16	20.00
	51 to 61	16	20.00
	62 to 72	9	11.25
	Above 72	2	2.50
**Age cutoff for upper endoscopy (years old)**	< 40	37	46.25
	≥ 40	43	53.75
**Signs and symptoms**	Epigastralgia	62	77.50
	Postprandial fullness	40	50.00
	Regurgitation	35	43.75
	Pyrosis	33	41.25
	Early satiety	27	33.75
	Nausea	26	32.50
	Sporadic vomiting	16	20.00
	Unexplained weight loss	14	17.50
	Dysphagia	7	8.75
	Gastrointestinal bleeding	5	6.25
	Globus pharyngeus	4	5.00
	Odynophagia	4	5.00
	Phantogeusia	1	1.25
	Diarrhea	1	1.25
**Number of signs and symptoms**	1	14	17.50
	2	19	23.75
	3	18	22.50
	4	13	16.25
	5	7	8.75
	6	2	2.50
	7	3	3.75
	8	4	5.00
**Indication for appropriate upper endoscopy**	Yes	57	71.25
	No	23	28.75
**Criteria for appropriate upper endoscopy**	Age ≥ 40 years old	43	53.75
	Symptoms refractory to treatment	14	17.50
	Unexplained weight loss	14	17.50
	Anemia	9	11.25
	Family history of gastric cancer	8	10.00
	Dysphagia	7	8.75
	Gastrointestinal bleeding	5	6.25
	Odynophagia	4	5.00
**Presence of risk factors**	Yes	64	80.00
	No	16	20.00
**Risk factors**	Coffee consumption	52	65.00
	Alcohol consumption	35	43.75
	Anti-inflammatory drug use	22	27.50
	Smoking	13	16.25
	Family history of gastric cancer	8	10.00
**Presence of comorbidities**	Yes	28	35.00
	No	52	65.00
**Comorbidities**	Hypertension	16	20.00
	Diabetes *mellitus*	11	13.75
	Lumbar disc herniation	2	2.50
	Coronary artery disease	1	1.25
	Chronic kidney disease	1	1.25
	Arthritis	1	1.25
	Osteoporosis	1	1.25
	Liver cirrhosis	1	1.25
	Systemic lupus erythematosus	1	1.25
	Familial hypercholesterolemia	1	1.25
	Major depressive disorder	1	1.25
	Breast cancer	1	1.25
**Previous upper endoscopy**	No	37	46.25
	Yes	43	53.75

Source: Elaborated by the authos.

Among the indication criteria, age 40 years old or older was the most prevalent, followed by symptoms refractory to treatment (17.5%) and unexplained weight loss (17.50%). Among all, 53.75% reported having undergone at least one previous UGE. Most patients had no comorbidities (65.82%), and among those who did, systemic arterial hypertension (20%) and type 2 diabetes *mellitus* (13.75%) were the most prevalent. Among the patients, 80% had risk factors, with coffee consumption being the most common (65%).

### Examination results

According to Table 2, mild enanthematous antral gastritis was the most prevalent finding (21.25%), followed by mild enanthematous pangastritis (12.50%), and moderate enanthematous pangastritis (11.25%). Regarding the number of lesions presented by patients, 75% had one lesion, 20% had two, and 5% had three. Among the patients, 10% had UGE without alterations. The lesion location was gastric in 85% of patients, and in 92.5% the alterations were not significant. The urease rapid test was positive in 25% of patients.

**Table 2:** Endoscopic findings of patients with dyspepsia undergoing upper endoscopy.

Variables		n	%
**Urease rapid test**	Negative	60	75
	Positive	20	25
**Upper endoscopy results**	Normal exams	8	10
	Organic lesion	72	90
**Anatomical location of the lesion**	Gastric lesion	68	85
	Esophageal lesion	13	16.25
	Duodenal lesion	7	8.75
**Severity of the lesion**	Not significant	74	92.5
	Significant	6	7.50
**Esophageal lesion**	Erosive esophagitis grade A Los Angeles	7	8.75
	Erosive esophagitis grade B Los Angeles	4	5.00
	Hiatal hernia grade 1	2	2.50
	Medium and large caliber esophageal varices	1	1.25
	Esophageal stenosis	1	1.25
	Barrett's esophagus	1	1.25
**Gastric lesion**	Mild enanthematous gastritis of the antrum	17	21.25
	Mild enanthematous pangastritis	10	12.50
	Moderate enanthematous pangastritis	9	11.25
	Moderate erosive gastritis of the antrum	7	8.75
	Mild erosive gastritis of the antrum	4	5.00
	Nodules in the antrum	4	5.00
	Mild pangastritis with erosions in the antrum	3	3.75
	Mild pangastritis with elevated erosive component in the antrum	1	1.25
	Mild pangastritis	1	1.25
	Moderate pangastritis with erosions in the antrum	1	1.25
	Mild erosive pangastritis	1	1.25
	Moderate erosive pangastritis	1	1.25
	Moderate enanthematous gastritis of the antrum	1	1.25
	Mild enanthematous gastritis of the body	1	1.25
	Mild enanthematous gastritis with mild erosive component of the antrum	1	1.25
	Moderate enanthematous gastritis of the body	1	1.25
	Mild elevated erosive gastritis of the antrum	1	1.25
	Mild erosive gastritis of the body	1	1.25
	Intense erosive gastritis of the antrum	1	1.25
	Intense elevated erosive gastritis of the antrum	1	1.25
	Isolated erosions in angular incisures	1	1.25
	Polyp in the gastric antrum	1	1.25
	Polyp of gastric antrum Paris 0 - IS	1	1.25
	Active gastric ulcer	1	1.25
	Antral sakita h2 ulcer	1	1.25
	Antral ulcer in the pre-pyloric sakita a2	1	1.25
**Duodenal lesion**	Moderate enanthematous bulboduodenitis	2	2.50
	Moderate erosive bulboduodenitis	2	2.50
	Sakita s1 duodenal ulcer	1	1.25
	Active duodenal ulcer	1	1.25
	Mild enanthematous bulboduodenitis	1	1.25
**Number of lesions per patient**	1	60	75.00
	2	16	20.00
	3	4	5.00

Source: Elaborated by the authos.

### Statistical correlations

According to Table 3, using Pearson and Fisher’s Exact correlation tests, there was a statistically significant correlation between age younger than 40 years old and a positive urease rapid test (*p* = 0.012), and female gender with a positive urease test (*p* = 0.041). There was no significant correlation between appropriate or inappropriate indication for UGE and urease test (*p* = 0.476).

**Table 3:** Correlation between clinical presentation and urease rapid test.

**Variables**		**Positive, n = 201 (%)**	**Negative, n = 601 (%)**	***p*-value**
**Sex**	Female	17 (85.00)	36 (60.00)	0.041
	Male	3 (15.00)	24 (40.00)	
**Age by age group (years old)**	18 to 28	4 (20.00)	4 (6.67)	0.365
	29 to 39	8 (40.00)	21 (35.00)	
	40 to 50	5 (25.00)	11 (18.33)	
	51 to 61	2 (10.00)	14 (23.33)	
	62 to 72	1 (5.00)	8 (13.33)	
	Over 72	0 (0.00)	2 (3.33)	
**Age ≥ 40 years old**	No	12 (60.00)	25 (41.70)	0.012
	Yes	8 (40.00)	35 (58.30)	
**Signs and symptoms**				
**Regurgitation**	No	8 (40.00)	37 (61.67)	0.091
	Yes	12 (60.00)	23 (38.33)	
**Heartburn**	No	9 (45.00)	38 (63.33)	0.149
	Yes	11 (55.00)	22 (36.67)	
**Epigastralgia**	No	3 (15.00)	15 (25.00)	0.538
	Yes	17 (85.00)	45 (75.00)	
**Postprandial fullness**	No	13 (65.00)	27 (45.00)	0.121
	Yes	7 (35.00)	33 (55.00)	
**Pharyngeal globus**	No	19 (95.00)	57 (95.00)	1.000
	Yes	1 (5.00)	3 (5.00)	
**Sporadic vomiting**	No	15 (75.00)	49 (81.67)	0.530
	Yes	5 (25.00)	11 (18.33)	
**Phantogeusia**	No	20 (100.00)	59 (98.33)	1.000
	Yes	0 (0.00)	1 (1.67)	
**Early satiety**	No	12 (60.00)	41 (68.33)	0.495
	Yes	8 (40.00)	19 (31.67)	
**Nausea**	No	11 (55.00)	43 (71.67)	0.168
	Yes	9 (45.00)	17 (28.33)	
**Diarrhea**	No	20 (100.00)	59 (98.33)	1.000
	Yes	0 (0.00)	1 (1.67)	
**Gastrointestinal bleeding**	No	19 (95.00)	57 (95.00)	1.000
	Yes	1 (5.00)	3 (5.00)	
**Dysphagia**	No	17 (85.00)	56 (93.30)	0.493
	Yes	3 (15.00)	4 (6.70)	
**Odynophagia**	No	19 (95.00)	57 (95.00)	1.000
	Yes	1 (5.00)	3 (5.00)	
**Unexplained weight loss**	No	17 (85.00)	49 (81.67)	1.000
	Yes	3 (15.00)	11 (18.33)	
**Indication for appropriate upper endoscopy**	No	7 (35.00)	16 (26.67)	0.476
	Yes	13 (65.00)	44 (73.33)	
**Endoscopy history**	No	12 (60.00)	25 (41.67)	0.154
	Yes	8 (40.00)	35 (58.33)	
**Risk factors**	No	3 (15.00)	9 (15.00)	1.000
	Yes	17 (85.00)	51 (85.00)	
**Alcohol consumption**	No	15 (75.00)	30 (50.00)	0.051
	Yes	5 (25.00)	30 (50.00)	
**Coffee consumption**	No	6 (30.00)	22 (36.67)	0.588
	Yes	14 (70.00)	38 (63.33)	
**Anti-inflammatory use**	No	15 (75.00)	43 (71.67)	0.772
	Yes	5 (25.00)	17 (28.33)	
**Smoking**	No	19 (95.00)	48 (80.00)	0.167
	Yes	1 (5.00)	12 (20.00)	
**Family history of gastric cancer**	No	19 (95.00)	53 (88.33)	0.672
	Yes	1 (5.00)	7 (11.67)	

* *p* calculated using Pearson χ^2^ test;

Fisher’s exact test. Source: Elaborated by the authors.

Table 4 describes the correlations between clinical data and the location of the lesion. Symptoms of pyrosis and regurgitation were related to esophageal lesions in only 12.8 (*p* = 0.711) and 17.5% (*p* = 0.724), respectively. A significant relationship was found between smoking and gastric lesions (*p* = 0.021). A normal endoscopic examination was significantly correlated with age younger than 40 years old (*p* = 0.022).

**Table 4:** Correlation between clinical presentation and anatomical site of lesion in upper endoscopy.

Variables		Gastric lesion, n = 681 (%)	Esophageal lesion, n = 131 (%)	Duodenal lesion, n = 71 (%)	Normal exam, n = 81 (%)	*p*-value
**Age ≥ 40 years old**	< 40 years	29 (42.65)	5 (38.46)	1 (14.29)	7 (87.50)	0.022
	≥ 40 years	39 (57.35)	8 (61.54)	6 (85.71)	1 (12.50)	
**Signs and symptoms**						
**Regurgitation**	No	39 (57.35)	6 (46.15)	7 (100.00)	4 (50.00)	0.724
	Yes	29 (42.65)	7 (53.85)	0 (0.00)	4 (50.00)	
**Heartburn**	No	42 (61.76)	8 (61.54)	3 (42.86)	4 (50.00)	0.711
	Yes	26 (38.24)	5 (38.46)	4 (57.14)	4 (50.00)	
**Epigastralgia**	No	12 (17.65)	3 (23.08)	1 (14.29)	3 (37.50)	0.370
	Yes	56 (82.35)	10 (76.92)	6 (85.71)	5 (62.50)	
**Postprandial fullness**	No	33 (48.53)	6 (46.15)	2 (28.57)	4 (50.00)	1.000
	Yes	35 (51.47)	7 (53.85)	5 (71.43)	4 (50.00)	
**Globus pharyngeus**	No	64 (94.12)	11 (84.62)	7 (100.00)	8 (100.00)	1.000
	Yes	4 (5.88)	2 (15.38)	0 (0.00)	0 (0.00)	
**Vomiting**	No	55 (80.88)	13 (100.00)	7 (100.00)	5 (62.50)	0.194
	Yes	13 (19.12)	0 (0.00)	0 (0.00)	3 (37.50)	
**Phantom taste**	No	67 (98.53)	13 (100.00)	7 (100.00)	8 (100.00)	1.000
	Yes	1 (1.47)	0 (0.00)	0 (0.00)	0 (0.00)	
**Early satiety**	No	43 (63.24)	10 (76.92)	6 (85.71)	7 (87.50)	0.255
	Yes	25 (36.76)	3 (23.08)	1 (14.29)	1 (12.50)	
**Nausea**	No	46 (67.65)	12 (92.31)	6 (85.71)	4 (50.00)	0.427
	Yes	22 (32.35)	1 (7.69)	1 (14.29)	4 (50.00)	
**Diarrhea**	No	67 (98.53)	13 (100.00)	7 (100.00)	8 (100.00)	1.000
	Yes	1 (1.47)	0 (0.00)	0 (0.00)	0 (0.00)	
**Gastrointestinal bleeding**	No	65 (95.59)	11 (84.62)	6 (85.71)	8 (100.00)	1.000
	Yes	3 (4.41)	2 (15.38)	1 (14.29)	0 (0.00)	
**Dysphagia**	No	63 (92.65)	13 (100.00)	7 (100.00)	6 (75.00)	0.143
	Yes	5 (7.35)	0 (0.00)	0 (0.00)	2 (25.00)	
**Odynophagia**	No	65 (95.59)	13 (100.00)	7 (100.00)	7 (87.50)	0.350
	Yes	3 (4.41)	0 (0.00)	0 (0.00)	1 (12.50)	
**Unexplained weight loss**	No	58 (85.29)	10 (76.92)	7 (100.00)	6 (75.00)	0.624
	Yes	10 (14.71)	3 (23.08)	0 (0.00)	2 (25.00)	
**Indication for appropriate upper endoscopy**	No	19 (27.94)	4 (30.77)	0 (0.00)	4 (50.00)	0.218
	Yes	49 (72.06)	9 (69.23)	7 (100.00)	4 (50.00)	
**Previous upper endoscopy**	No	32 (47.06)	4 (30.77)	4 (57.14)	5 (62.50)	0.461
	Yes	36 (52.94)	9 (69.23)	3 (42.86)	3 (37.50)	
**Presence of risk factors**	No	10 (14.71)	0 (0.00)	2 (28.57)	1 (12.50)	1.000
	Yes	58 (85.29)	13 (100.00)	5 (71.43)	7 (87.50)	
**Alcohol consumption**	No	40 (58.82)	4 (30.77)	5 (71.43)	3 (37.50)	0.288
	Yes	28 (41.18)	9 (69.23)	2 (28.57)	5 (62.50)	
**Coffee consumption**	No	23 (33.82)	2 (15.38)	3 (42.86)	4 (50.00)	0.441
	Yes	45 (66.18)	11 (84.62)	4 (57.14)	4 (50.00)	
**Use of anti-inflammatory drugs**	No	47 (69.12)	9 (69.23)	6 (85.71)	7 (87.50)	0.434
	Yes	21 (30.88)	4 (30.77)	1 (14.29)	1 (12.50)	
**Smoking**	No	59 (86.76)	11 (84.62)	4 (57.14)	4 (50.00)	0.021
	Yes	9 (13.24)	2 (15.38)	3 (42.86)	4 (50.00)	
**Family history of gastric cancer**	No	61 (89.71)	12 (92.31)	6 (85.71)	7 (87.50)	0.587
	Yes	7 (10.29)	1 (7.69)	1 (14.29)	1 (12.50)	

Source: Elaborated by the authors.

All patients with significant findings had appropriate indications for UGE (*p* = 0.175) (Table 5).

**Table 5:** Correlation between clinical presentation and severity of lesions in upper endoscopy.

Variables		Non-significant, n = 741	Significant, n = 61	*p*-value
**Age ≥ 40 years old**	No	35 (47.30)	2 (33.33)	0.681
	Yes	39 (52.70)	4 (66.67)	
**Signs and symptoms**				
**Regurgitation**	No	40 (54.05)	5 (83.33)	0.223
	Yes	34 (45.95)	1 (16.67)	
**Pyrosis**	No	44 (59.46)	3 (50.00)	0.687
	Yes	30 (40.54)	3 (50.00)	
**Epigastralgia**	No	17 (22.97)	1 (16.67)	1.000
	Yes	57 (77.03)	5 (83.33)	
**Postprandial fullness**	No	37 (50.00)	3 (50.00)	1.000
	Yes	37 (50.00)	3 (50.00)	
**Pharyngeal bolus**	No	70 (94.59)	6 (100.00)	1.000
	Yes	4 (5.41)	0 (0.00)	
**Vomiting**	No	59 (79.73)	5 (83.33)	1.000
	Yes	15 (20.27)	1 (16.67)	
**Phantogeusia**	No	73 (98.65)	6 (100.00)	1.000
	Yes	1 (1.35)	0 (0.00)	
**Early satiety**	No	47 (63.51)	6 (100.00)	0.092
	Yes	27 (36.49)	0 (0.00)	
**Nausea**	No	51 (68.92)	3 (50.00)	0.384
	Yes	23 (31.08)	3 (50.00)	
**Diarrhea**	No	73 (98.65)	6 (100.00)	1.000
	Yes	1 (1.35)	0 (0.00)	
**Gastrointestinal bleeding**	No	72 (97.30)	4 (66.67)	0.027
	Yes	2 (2.70)	2 (33.33)	
**Dysphagia**	No	67 (90.54)	6 (100.00)	1.000
	Yes	7 (9.46)	0 (0.00)	
**Odynophagia**	No	70 (94.59)	6 (100.00)	1.000
	Yes	4 (5.41)	0 (0.00)	
**Unexplained weight loss**	No	61 (82.43)	5 (83.33)	1.000
	Yes	13 (17.57)	1 (16.67)	
**Refractory symptoms**	No	62 (83.78)	4 (66.67)	0.281
	Yes	12 (16.22)	2 (33.33)	
**Presence of indication for appropriate upper endoscopy**	No	23 (31.08)	0 (0.00)	0.175
	Yes	51 (68.92)	6 (100.00)	
**Previous upper endoscopy**	No	33 (44.59)	4 (66.67)	0.407
	Yes	41 (55.41)	2 (33.33)	
**Risk factors**				
**Alcohol abuse**	No	41 (55.41)	4 (66.67)	0.691
	Yes	33 (44.59)	2 (33.33)	
**Coffee abuse**	No	25 (33.78)	3 (50.00)	0.417
	Yes	49 (66.22)	3 (50.00)	
**Non-steroidal anti-inflammatory abuse**	No	52 (70.27)	6 (100.00)	0.180
	Yes	22 (29.73)	0 (0.00)	
**Smoking**	No	62 (83.78)	5 (83.33)	1.000
	Yes	12 (16.22)	1 (16.67)	
**Family history of gastric cancer**	No	67 (90.54)	5 (83.33)	0.480
	Yes	7 (9.46)	1 (16.67)	
**Comorbidities**	No	46 (63.89)	4 (66.67)	1.000
	Yes	26 (36.11)	2 (33.33)	
**Diabetes mellitus**	No	64 (86.49)	5 (83.33)	1.000
	Yes	10 (13.51)	1 (16.67)	
**Systemic arterial hypertension**	No	60 (81.08)	4 (66.67)	0.594
	Yes	14 (18.92)	2 (33.33)	
**Coronary artery disease**	No	73 (98.65)	6 (100.00)	1.000
	Yes	1 (1.35)	0 (0.00)	
**Chronic kidney disease**	No	74 (100.00)	5 (83.33)	0.075
	Yes	0 (0.00)	1 (16.67)	
**Arthritis**	No	73 (98.65)	6 (100.00)	1.000
	Yes	1 (1.35)	0 (0.00)	
**Osteoporosis**	No	73 (98.65)	6 (100.00)	1.000
	Yes	1 (1.35)	0 (0.00)	
**Hepatic cirrhosis**	No	73 (98.65)	6 (100.00)	1.000
	Yes	1 (1.35)	0 (0.00)	
**Lumbar disc herniation**	No	72 (97.30)	6 (100.00)	1.000
	Yes	2 (2.70)	0 (0.00)	
**Systemic lupus erythematosus**	No	73 (98.65)	6 (100.00)	1.000
	Yes	1 (1.35)	0 (0.00)	
**Familial hypercholesterolemia**	No	73 (98.65)	6 (100.00)	1.000
	Yes	1 (1.35)	0 (0.00)	
**Major depressive disorder**	No	73 (98.65)	6 (100.00)	1.000
	Yes	1 (1.35)	0 (0.00)	

Source: Elaborated by the authors.

## Discussion

Regarding the variable of gender in the patients analyzed in this study, there was compatibility with a study conducted in Belém, PA, Brazil, by Domingues et al.^9^, which indicated a higher prevalence of females (65%). A systematic review of gender-based prevalence studies showed inconsistent prevalence of dyspepsia in men and women^10^. Thus, we postulate that women are more likely to seek medical attention for dyspepsia symptoms than men, explaining the higher number of endoscopies performed on female patients^11^.

Regarding the age of the patients who underwent the examination, Batool et al.^12^ found that 67% were in the age group > 40 years old, a higher value than the 53.75% found in this study. However, these findings are in line with some guidelines for UGE indication, such as the IV Brazilian Consensus on *H. pylori* Infection^4^. In addition, the upper gastrointestinal tract symptomatology presented by the patients is in line with Serra et al.^13^, who demonstrated epigastric pain as the most prevalent (83%).

Regarding the appropriate or inappropriate indication of patients for UGE, there was variation in the literature depending on which guidelines the articles followed. In this study, 29.75% of patients were inappropriately indicated according to established criteria. In this context, the study by Meira et al.^14^, which used American Society for Gastrointestional Endoscopy criteria, and the study by Gupta et al.^15^, using criteria from the American College of Gastroenterology and the Canadian Association of Gastroenterology, found 39.5 and 75.5%, respectively, of inappropriate indications. Furthermore, a systematic review by Hassan and Zullo^16^, which included 23 studies and 53,392 patients, showed that UGE was inappropriately indicated in 21.7% of cases, and, despite a decline in inappropriate indications during the period analyzed by the authors, this rate is still higher than 20%, which is in line with the present research.

The majority of analyzed patients had undergone previous endoscopic examinations (53.75%), which is in agreement with findings in the analyzed literature, such as the study by Meira et al.^14^, in which this percentage was 61.4%. In this context, this fact may be related to the chronic nature of gastrointestinal diseases and the need for multifactorial treatment for better effectiveness, such as lifestyle changes^17^.

The data on patients with comorbidities (35%) are in line with those found by Meira et al.^14^ (38%), demonstrating that the minority of dyspeptic patients had comorbidities. These facts can be explained by comparing the small number of patients (13.75%) aged 62 years old or older in this study and in Silva and Breda’s study^18^, which showed a higher prevalence of comorbidities in elderly patients. Furthermore, regarding risk factors, daily coffee consumption in the Japanese study by Haruma et al.^17^ was 32.9% of patients and lower than that found in this study, reflecting this common habit in Brazil and the high number of patients with risk factors.

The percentage of normal endoscopic reports was higher than indicated in the national literature. Normal endoscopic reports were higher (10%) compared to national literature. Domingues et al.^9^, in a study conducted in the state of Pará, and Rolim Junior et al.^19^, in Sergipe, Brazil, found a prevalence of normal exams of 1.8 and 6.5%, respectively. This can be explained by analyzing whether the indication was appropriate or not for the exam, such as Keren et al.’s study^3^, which found that a normal endoscopic finding was less frequent when American Society for Gastrointestinal Endoscopy indications were followed (*p* < 0.001).

The anatomical location of the lesion in UGE is in accordance with Rolim Junior et al.’s study^19^, who found gastric lesions as the main location in 64%. Regarding the most prevalent finding in the UGE report, this study found that mild enanthematous gastritis of the antrum was present in 21.25% of patients and is in accordance with Domingues et al.’s study^9^, but in a lower proportion, as they found 72.9% of people with enanthematous antral gastritis. Such discrepancies in values can be explained by the classification made by these authors, as they grouped all enanthematous antral gastritis, without specifying if it was mild or moderate.

Regarding significant findings (7.5%), the data found are similar to the recent study by Abdeljawad et al.^20^, which found 10.2%. However, they are lower than those found by Wallace et al.^7^ (21%), an older study. We postulate that these results are lower due to the easier access to medications nowadays, the widespread use of PPIs in clinical practice, and the reduced prevalence of *H. pylori* infection^21^.

Regarding the result of the positive urease rapid test (25%), there was variation in prevalence when compared to national literature. In this sense, Vaz et al.^22^ found 44.54% positive tests in patients undergoing UGE in Itabirito, MG, Brazil, and Frugis et al.^23^, in a study conducted in São Paulo, SP, Brazil, found 17% positive results in a retrospective analysis of 10 years of patients undergoing UGE. These factors can be explained by the great variation in national territory of risk factors related to *H. pylori* infection, such as poor home conditions and low educational levels^24^.

Regarding the correlation between the symptoms presented by patients and the anatomical locations of lesions in UGE, although it did not show statistically significant correlation, symptoms of heartburn and regurgitation were related to esophageal lesions in only 12.8 (*p* = 0.711) and 17.5% (*p* = 0.724), respectively. These data are interesting to reaffirm that the diagnosis of GERD is clinical, since in up to 70% of cases it is non-erosive reflux disease, in which there are no signs of mucosal damage via endoscopy^25^. Furthermore, a significant relationship was found between smoking and gastric lesions (*p* = 0.021), reaffirming the involvement of smoking in peptic lesions, such as gastritis, through various mechanisms, including vasoconstriction in the mucosa^17^.

Normal endoscopic examination was significantly correlated with age under 40 years old (*p* = 0.022). In this context, these data are in line with the study by Batool et al.^12^, which found a significant finding between age over 40 years old and a higher incidence of organic dyspepsia (*p* <0.01).

Although there was no statistically significant association between significant findings and appropriate UGE indication criteria (*p* = 0.175), all patients with significant findings had appropriate indications for UGE. In this context, the study by Abdeljawad et al.20 found an association between significant findings and alarm signs, demonstrating the importance of these signs in identifying more serious pathologies in patients. Additionally, the study by Crouwel et al..26 suggested that patients under 40 years old can be treated without UGE, with a very low risk of missing a curable malignancy. On the other hand, these data demonstrate the low frequency of significant findings in patients without appropriate indications for UGE. In this regard, due to the high prevalence of dyspepsia, an immediate endoscopy for each dyspeptic patient is not a practical approach, as this will lead to high costs and low endoscopy yield^20^.

A significant relationship was found between age under 40 and positive urease rapid test (*p* = 0.012). These values are in line with those found by Singh et al.^27^, who found a high incidence of *H. pylori* infection in patients aged 20 to 30 years old and a decreasing incidence in the age group above 50 years old (*p* = 0.0242). That said, studies show that *H. pylori* infection in the elderly is easier to eradicate, as there is atrophy of the stomach mucosa with aging, making colonization by the bacteria unviable^28^.

Furthermore, when we relate gender to urease, there was a statistically significant relationship between female gender and positive urease (*p* = 0.041). In this context, these values differ from those found by R. M. and Shashidhara^29^, who demonstrated a higher prevalence of positive results in males. The explanation for this could be verified by the higher number of women in the current research and a higher prevalence of men in comparative studies, interfering with the results. Thus, when comparing with studies by Vaz et al.^22^ and Frugis et al.^23^, which had more women in their research, there was a higher prevalence of positive urease rapid test results in females, 66.32 and 59.25%, respectively.

Moreover, although there was no statistically significant relationship between patients with appropriate or inappropriate indication for UGE and urease test (*p* = 0.476), 35% of positive urease results were from patients inappropriately indicated for UGE. Thus, according to various guidelines, when the patient does not meet the criteria for appropriate UGE indication, the test and treat strategy should be employed with non-invasive *H. pylori* tests, such as the 13C-urea breath test, which is more cost-effective and reduces the demand for UGE. However, this test has not yet been incorporated into daily clinical practice in Brazil due to national authorities’ restrictions on the substrate^4^. Thus, this lack of non-invasive alternatives for *H. pylori* diagnosis drives the need for UGE in young dyspeptic patients without alarm signs^30^. This fact implies, for example, whether the criteria for appropriate UGE indication should be reformulated in Brazil or there is a need for greater encouragement of non-invasive *H. pylori* tests.

## Conclusion

The study allowed us to assert that most patients undergoing UGE due to symptoms in the interior of Maranhão were female, aged 40 or older, presenting more than two symptoms, with epigastric pain being the main one. Many did not have a proper indication for UGE according to the criteria of the IV Brazilian Consensus Conference on *H. pylori* Infection. Most had no comorbidities, and many had risk factors. Furthermore, the main finding in UGE was mild enanthematous gastritis of the antrum, the stomach being the main site affected, and the majority was non-significant alterations. The rapid urease test had results that differ from other regions of Brazil, with a relationship between age under 40 and female gender with a positive urease result. Finally, smoking was related to gastric lesions, and a normal endoscopic exam was related to age under 40.

## Data Availability

All data sets were generated or analyzed in the current study;

## References

[1] Colin-Jones DG, Bloom B, Bodemar G (1988). Management of dyspepsia: Report of working party. Lancet.

[2] Moayyedi PM, Lacy BE, Andrews CN, Enns RA, Howden CW, Vakil N (2017). ACG and CAG clinical guideline: management of dyspepsia. Am J Gastroenterol.

[3] Keren D, Rainis T, Stermer E, Lavy A (2011). A nine-year audit of open-access upper gastrointestinal endoscopic procedures: results and experience of a single centre. Can J Gastroenterol.

[4] Coelho LGV, Marinho JR, Genta R, Ribeiro LT, Passos MDC, Zaterka S, Assumpção PP, Barbosa AJA, Barbuti R, Braga LL, Breyer H, Carvalhaes A, Chinzon D, Cury M, Domingues G, Jorge JL, Maguilnik I, Marinho FP, Moraes-Filho JP, Paula-E-Silva CM, Pedrazzoli-Júnior J, Ramos AFP, Seidler H, Spinelli JN, Zir JV (2018). IVth Brazilian consensus conference on *Helicobacter pylori* infection. Arq Gastroenterol.

[5] Mahadeva S, Goh KL (2006). Epidemiology of functional dyspepsia: a global perspective. World J Gastroenterol.

[6] Peery AF, Crockett SD, Murphy CC, Lund JL, Dellon ES, Williams JL, Jensen ET, Shaheen NJ, Barritt AS, Lieber SR, Kochar B, Barnes EL, Fan YC, Pate V, Galanko J, Baron TH, Sandler RS (2019). Burden and cost of gastrointestinal, liver, and pancreatic diseases in the United States: update 2018. Gastroenterology.

[7] Wallace MB, Durkalski VL, Vaughan J, Palesch YY, Libby ED, Jowell PS, Nickl NJ, Schutz SM, Leung JW, Cotton PB (2001). Age and alarm symptoms do not predict endoscopic findings among patients with dyspepsia: a multicentre database study. Gut.

[8] Oliveira SSD, Santos IDSD, Silva JFPD, Machado EC (2006). Prevalência de dispepsia e fatores sociodemográficos. Rev Saúde Pública.

[9] Domingues EP, Rymsza GP, Caldato C, de Melo L, de Oliveira ACM (2023). Avaliação dos resultados dos exames de Endoscopia Digestiva Alta realizadas em um hospital do município de Belém-PA no ano de 2019. Rev Eletr Acervo Saúde.

[10] Ahlawat SK, Cuddihy MT, Locke GR (2006). Gender-related differences in dyspepsia: a qualitative systematic review. Gend Med.

[11] Koloski NA, Talley NJ, Boyce PM (2002). Epidemiology and health care seeking in the functional GI disorders: a population-based study. Am J Gastroenterol.

[12] Batool S, Sajid MU, Waris J, Satti SA, Hassan F, Aziz F (2021). Endoscopic findings in patients presenting with dyspepsia and association of age and gender with organic dyspepsia. Pak Armed Forces Med J.

[13] Serra MAA, Medeiros AT, Torres MD, Dias ICC, Santos CAA, Araújo MD (2021). Correlation between the symptoms of upper gastrointestinal disease and endoscopy findings: Implications for clinical practice. J Taibah Univ Med Sci.

[14] Meira ATDS, Tanajura D, Viana ISS (2019). Clinical and endoscopic evaluation in patients with gastroesophageal symptoms. Arq Gastroenterol.

[15] Gupta K, Groudan K, Jobbins K, Hans B, Singhania R (2021). Single-center review of appropriateness and utilization of upper endoscopy in dyspepsia in the United States. Gastroenterol Res.

[16] Zullo A, Manta R, De Francesco V, Fiorini G, Hassan C, Vaira D (2019). Diagnostic yield of upper endoscopy according to appropriateness: A systematic review. Dig Liver Dis.

[17] Haruma K, Kinoshita Y, Sakamoto S, Sanada K, Hiroi S, Miwa H (2015). Lifestyle factors and efficacy of lifestyle interventions in gastroesophageal reflux disease patients with functional dyspepsia: primary care perspectives from the LEGEND study. Intern Med.

[18] Silva LB, Breda D (2023). A prevalência e distribuição de comorbidades envolvidas nos pacientes idosos em uma Unidade de Saúde do município de Cascavel-PR. Res Soc Dev.

[19] Rolim RAS, Barreto ASM, de Carvalho Nascimento E, de Lima Mota M, Lima FS, Soares ACGM (2021). Prevalência dos achados endoscópicos em Sergipe. Res Soc Dev.

[20] Abdeljawad K, Wehbeh A, Qayed E (2017). Low prevalence of clinically significant endoscopic findings in outpatients with dyspepsia. Gastroenterol Res Pract.

[21] Scarpignato C, Gatta L, Zullo A, Blandizzi C, SIF-AIGO-FIMMG Group; Italian Society of Pharmacology, the Italian Association of Hospital Gastroenterologists, and the Italian Federation of General Practitioners (2016). Effective and safe proton pump inhibitor therapy in acid-related diseases–A position paper addressing benefits and potential harms of acid suppression. BMC Med.

[22] Vaz AFC, Belarmino DAA, Ferreira JGG, Frade RI, Silva RO (2021). Prevalência de infecção por *Helicobacter Pylori* em pacientes submetidos à endoscopia digestiva alta do Centro de Especialidades Médicas da cidade de Itabirito/MG. NBC-Periódico Cient do Núcleo de Biociências.

[23] Frugis S, Czeczko NG, Malafaia O, Parada AA, Poletti PB, Secchi TF, Degiovani M, Rampanazzo-Neto A, D’Agostino MD (2016). Prevalência do *helicobacter pylori* há dez anos comparada com a atual em pacientes submetidos à endoscopia digestiva alta. ABCD. Arq Bras Cir Dig.

[24] Moosazadeh M, Lankarani KB, Afshari M (2016). Meta-analysis of the prevalence of *Helicobacter pylori* infection among children and adults of Iran. Int J Prev Med.

[25] Quigley EM (2001). Non-erosive reflux disease: part of the spectrum of gastro-oesophageal reflux disease, a component of functional dyspepsia, or both?. Eur J Gastroenterol Hepatol.

[26] Crouwel F, Meurs-Szojda MM, Klemt-Kropp M, Fockens P, Grasman ME (2018). The diagnostic yield of open-access endoscopy of the upper gastrointestinal tract in the Netherlands. Endoscopy Int Open.

[27] Singh SKR, Kamendu A, Kishor A (2020). A study on the prevalence of *helicobacter pylori* infection by rapid urease test in patients undergoing upper gastro intestinal endoscopy for dyspepsia in a tertiary care hospital of Southern Bihar. Int J Health Clin Res.

[28] Israel DA, Peek RM (2001). Pathogenesis of *Helicobacter pylori*-induced gastric inflammation. Aliment Pharmacol Ther.

[29] RM SS, Shashidhara P (2022). Prevalence of *Helicobacter pylori* infection by rapid urease test among patients with dyspeptic symptoms who underwent upper gastrointestinal endoscopy in a secondary care hospital. Int Surg J.

[30] Cheddie S, Manneh CG, Owczarek BM, Moodley Y (2020). Age is a predictor of significant endoscopic findings in dyspepsia patients in South Africa. S Afr J Surg.

